# Maternal Depletion of Piwi, a Component of the RNAi System, Impacts Heterochromatin Formation in *Drosophila*

**DOI:** 10.1371/journal.pgen.1003780

**Published:** 2013-09-19

**Authors:** Tingting Gu, Sarah C. R. Elgin

**Affiliations:** Department of Biology, Washington University, Saint Louis, Missouri, United States of America; Institute of Molecular Biotechnology (IMBA), United States of America

## Abstract

A persistent question in epigenetics is how heterochromatin is targeted for assembly at specific domains, and how that chromatin state is faithfully transmitted. Stable heterochromatin is necessary to silence transposable elements (TEs) and maintain genome integrity. Both the RNAi system and heterochromatin components HP1 (Swi6) and H3K9me2/3 are required for initial establishment of heterochromatin structures in *S. pombe*. Here we utilize both loss of function alleles and the newly developed *Drosophila melanogaster* transgenic shRNA lines to deplete proteins of interest at specific development stages to dissect their roles in heterochromatin assembly in early zygotes and in maintenance of the silencing chromatin state during development. Using reporters subject to Position Effect Variegation (PEV), we find that depletion of key proteins in the early embryo can lead to loss of silencing assayed at adult stages. The piRNA component Piwi is required in the early embryo for reporter silencing in non-gonadal somatic cells, but knock-down during larval stages has no impact. This implies that Piwi is involved in targeting HP1a when heterochromatin is established at the late blastoderm stage and possibly also during embryogenesis, but that the silent chromatin state created is transmitted through cell division independent of the piRNA system. In contrast, heterochromatin structural protein HP1a is required for both initial heterochromatin assembly and the following mitotic inheritance. HP1a profiles in *piwi* mutant animals confirm that Piwi depletion leads to decreased HP1a levels in pericentric heterochromatin, particularly in TEs. The results suggest that the major role of the piRNA system in assembly of heterochromatin in non-gonadal somatic cells occurs in the early embryo during heterochromatin formation, and further demonstrate that failure of heterochromatin formation in the early embryo impacts the phenotype of the adult.

## Author Summary

Most eukaryotes harbor a high proportion of transposable elements (TEs) in their genomes. Heterochromatin, a condensed chromatin state found at domains enriched for TEs and other repetitious elements, is important for silencing TEs and maintaining the integrity of the genome. The RNAi system has been shown to be important for the establishment and maintenance of heterochromatin in both fungi and plants. To investigate whether this mechanism is also utilized in animals, we selectively depleted the proteins of interest in different developmental stages of *Drosophila melanogaster*, the fruit fly. Observing reporters subject to Position Effect Variegation (silencing due to heterochromatin formation), and using chromatin immunoprecipitation to establish heterochromatin protein levels at these sites, we find that the piRNA component Piwi is important for heterochromatin establishment during embryogenesis, but not for maintenance of the chromatin state in somatic cells. In contrast, the heterochromatin structural protein HP1a is essential for both establishment and maintenance. This suggests that RNAi components (specifically Piwi) play a guiding role in heterochromatin structure formation during embryogenesis, but have a minor role in maintenance. The findings emphasize the importance of maternal materials for the development of the *Drosophila* offspring, and demonstrate that failure of heterochromatin formation in the embryo impacts the adult phenotype.

## Introduction

Eukaryotic genomes are packaged into chromatin, which can broadly be characterized as having two alternative forms, euchromatin and heterochromatin. Heterochromatin was first distinguished as dense (darkly staining) chromosomal material, seen by microscopy [1]. Since that time, heterochromatin has been investigated extensively in systems from yeast to human to understand its characteristics and its biological significance. Euchromatin is gene-rich, and generally more accessible for transcription, while heterochromatin is gene poor, more condensed and exhibits highly regular nucleosome arrays [2], [3]. More recently, genome-wide mapping has shown that chromatin consists of numerous different states characterized by different patterns of histone modifications and associated chromosomal proteins [4]–[6]. Euchromatin is enriched in histone acetylation and H3K4me2/3, marks associated with active transcription; heterochromatin is typically enriched in silencing marks such as H3K9me2/3, and in heterochromatin protein HP1a [5].

While it is transcriptionally inert compared to euchromatin, heterochromatin plays an important role in a variety of biological processes, including regulation of DNA repair [7], [8], maintaining silencing of transposable elements (TEs), and maintaining the integrity of the genome [9]. Mis-regulation of the constituent proteins or regulators of heterochromatin formation will lead to malfunction of the cell, including development of cancers [10].

How the cell decides which regions of the genome to package as heterochromatin, with concomitant gene silencing, is an important question. Studies from diverse systems have indicated a role for non-coding RNA (ncRNA) in heterochromatin assembly. In *Schizosaccharomyces pombe*, the heterochromatic region surrounding centromeres contains *dg*/*dh* repeats, which are actively transcribed during S phase and believed to be the source of siRNAs [11], [12]. The siRNAs produced guide the RNA-induced transcriptional silencing (RITS) complex to the regions to be heterochromatized, resulting in localization of histone methyltransferase Clr4 to create methylated histone 3 lysine 9 (H3K9me2/3). This further stabilizes the RITS complex and leads to binding of the HP1a homolog Swi6 via its chromodomain, resulting in the spread of H3K9me2/3 enriched heterochromatin [12]. Similar RNA-associated heterochromatin targeting mechanisms have also been observed in plants, ciliates, worms (*C. elegans*), mammals and flies (*Drosophila*) [13]–[19]. In *Drosophila*, Piwi is the only one of five argonaute proteins (capable of binding small RNAs) that both enters the nucleus and plays a major role in silencing TEs. Hence it is considered a likely candidate to play a role in heterochromatin formation [3], [19]–[21].

Epigenetic signals are responsible for enabling the cells to “remember” the past stimulus, to sustain the chromatin states and transcriptional status that results [21]–[23]. Once established, whether the chromatin states (specifically histone modification patterns) can be inherited following mitosis, or whether they depend on a recurrent stimulus from the *cis*-element, is largely unknown. Some studies suggest that sequence specific elements and the RNA systems involved are required for maintenance of heterochromatin status [24]. However, recent work in yeast and worms suggests that heritable gene expression states and structural heterochromatin (H3K9me2/3) can be maintained in the absence of *cis* nucleating sequences or the original stimulus [25]–[27]. *Drosophila melanogaster*, a multicellular system with complex development, is a good choice for investigating this question.

*D. melanogaster*, a model organism used for genetic studies for over 100 years, has been an excellent system for the study of chromatin. Of its 180 Mb genome, about one third is packaged into heterochromatin [28]. A euchromatic gene juxtaposed to a heterochromatin mass, by rearrangement or transposition, exhibits stochastic expression in different cells, so called position effect variegation (PEV) [29], [30]. The silencing of the target gene (the reporter) in some cells in which it is normally active is believed to reflect the spreading of the silencing components from the adjacent heterochromatin mass; thus PEV is a sensitive reporter of the heterochromatic environment [19], [31], [32]. Screens for suppressors or enhancers of PEV have identified a variety of histone modifiers, chromatin structural components and other chromatin regulators [33], [34]. The central heterochromatin components, such as Heterochromatin Protein 1 (HP1a) and the histone H3K9 methyltransferase (HMTase) SU(VAR)3–9, are found in various species from yeast, to fruit fly, to mammals [35], [36], demonstrating the well-conserved mechanisms and evolutionary significance of heterochromatin in eukaryotes.

In *Drosophila melanogaster*, constitutive pericentric heterochromatin is not observed cytologically in the initial zygote, but emerges during blastoderm formation (∼2 hour embryo) [31], [37]. At this stage, how heterochromatin is established, whether the RNAi system is involved etc, is not clear. Chromatin assembly during this period (prior to significant zygotic transcription) is dependent on maternally loaded RNA and protein products, complicating genetic analysis. Analysis using an inducible *lacZ* reporter has found that silencing occurs at the onset of gastrulation, ∼1 hour after heterochromatin is visible cytologically [38]. Further investigation is needed to determine how the heterochromatin state is transmitted during development. To test whether the RNAi system and other heterochromatin components participate in initiation of heterochromatin formation in the early embryo and in the maintenance of heterochromatin during development, we knocked down the expression of those proteins of interest in different stages to dissect their possible roles. Using a combination of PEV and Chromatin Immuno-Precipitation (ChIP) assays, we find that maternal depletion of piRNA component Piwi, or of heterochromatin structural protein HP1a, results in a loss of reporter silencing in the adult, indicating that these proteins are actively involved in heterochromatin assembly in the early zygote; depletion of Piwi is associated with depletion of HP1a at the reporter site. In contrast, in post-gastrulation developing mitotic cells, only depletion of HP1a or H3K9 histone methyltransferase EGG, but not of RNAi components Piwi or AGO2, leads to loss of reporter silencing. ChIP-array data for HP1a profiles in the *piwi* mutant confirm that the HP1a level decreases at TE sequences in response to the depletion of Piwi. Our results suggest that the RNAi system plays a critical role in heterochromatin establishment and associated reporter silencing at the heterochromatin/euchromatin border, but plays a minor role in sustaining the chromatin states during development in *Drosophila*.

## Results

### 1. Depletion of functional HP1a in embryos leads to a long-lasting impact on PEV reporters seen in adult animals

**Figure 1:**
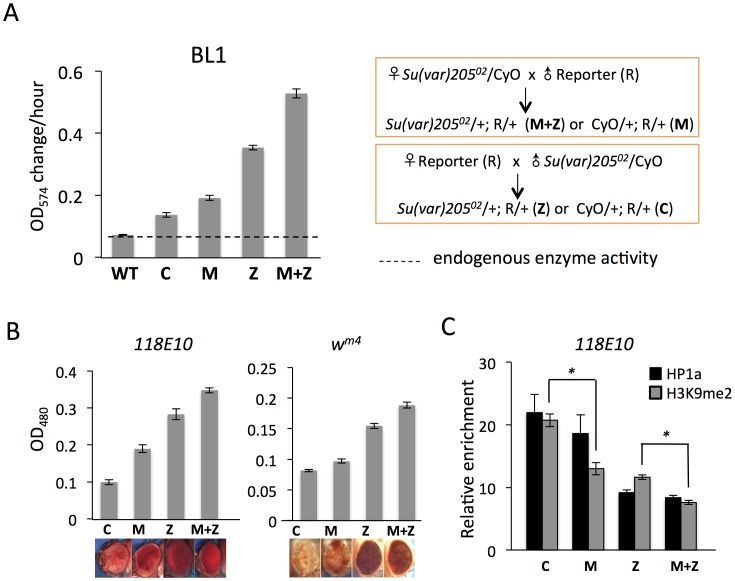
Depletion of functional HP1a in embryos leads to a long-lasting impact on chromatin structure in adult animals. (A) Quantitative β-galactosidase assays show that reduction of maternally loaded functional HP1a in embryos causes suppression of variegation of the *hsp70-lacZ* reporter on chromosome 3L (BL1). The crosses used to achieve depletion are shown on the right. Data from adult female flies. (B) Pigment assays and eye pictures show the suppression of variegation effect of maternal depletion of functional HP1a on the *hsp70-w* reporter *118E10* on chromosome 4, and on *w^m4^* (inversion on chromosome X). Data from adult female flies. (C) HP1a and H3K9me2 enrichment levels in the promoter region of the *118E10* reporter assayed by ChIP-qPCR. Data from mixed adult flies (both males and females). The HP1a enrichment level is normalized to the α-actinin locus. Error bars denote standard error of the mean (SEM). Note that the CyO balancer does not impact variegation of the BL1, *118E10* or *w^m4^* reporters in this genetic background (Figure S1). WT  =  wild type; C  =  control; M  =  maternal depletion; Z  =  zygotic depletion; M+Z  =  maternal and zygotic depletion.

HP1a is a critical protein for heterochromatin, thought to be required both for initial assembly and maintenance of heterochromatin structure. Silencing of PEV reporters is known to be sensitive to the dosage of HP1a [39]. While very high levels of expression of the HP1a gene [*Su(var)205*] are seen in the ovary and in the 0–12 hr embryo, the gene is expressed at moderately high levels throughout the life of the organism [40]. To test the effect of HP1a loss of function in early zygotes on heterochromatin structure in mature stages, we utilized HP1a mutant allele *Su(var)205^02^*, coupled with PEV reporters. The *Su(var)205^02^* loss of function allele has a V26M substitution that alters the chromo domain binding pocket, resulting in a loss of HP1a binding to H3K9me2/3. Given a wild type father, those offspring inheriting the *Su(var)205* wild type allele from *Su(var)205^02^*/+ heterozygous mothers experience only maternal depletion of HP1a (denoted as M in Figure 1), while the *Su(var)205^02^*/+ offspring experience both maternal and zygotic depletion (denoted as M+Z, Figure 1). Note that maternal depletion will impact both events in the female germ line and in the early zygote, based on maternal loading of chromosomal proteins and mRNAs. The reciprocal cross allows us to assay flies with depletion of HP1a in the paternal germ cells (thought to have minimal impact, so denoted C in Figure 1) and in the developing zygote only (Z, Figure 1). This experiment makes it possible to differentiate between the effects of maternal and zygotic functional HP1a depletion.

Using PEV reporter line BL1 (an inversion allele of the *hsp70*-*lacZ* transgenic reporter, with the reporter gene positioned adjacent to a 3L pericentric heterochromatin mass [41]), we find that this HP1a mutation leads to a significant increase in *hsp70*-*lacZ* expression in the adult fly. In M and Z flies, the β-galactosidase activity is increased by about 2- and 4-fold, respectively (Figure 1A); this suppression of PEV is additive, as shown by the ∼6-fold increase in M+Z flies (Figure 1A). Similarly, using the *hsp70-w* reporter *118E10* on chromosome 4 or the classic PEV reporter *w^m4^* (an inversion on the X chromosome), we find that the pigment levels of M, Z and M+Z flies successively increase, reflecting a more accessible chromatin structure at this locus when functional HP1a is reduced either in early zygotes or in developing animals (Figure 1B). This maternal effect is observed in both male and female flies (Figure 1, Figure S2). ChIP-qPCR (Chromatin Immuno-Precipitation – quantitative PCR) experiments in adult flies were performed to measure the enrichment levels of silencing marks HP1a and H3K9me2 at the *118E10* reporter to explore the perturbation of local chromatin structure. Indeed, both HP1a and H3K9me2 enrichment are decreased at the *hsp70* promoter sequence of the reporter in M, Z, and M+Z animals (Figure 1C), consistent with the expectations based on the eye pigment levels. In particular, the H3K9me2 level in C flies is significantly higher than that in M flies, and that in Z flies is significantly higher than that in M+Z flies (P<0.05, Figure 1C), indicating that maternal depletion of functional HP1a, which impacts the early zygote at the time of heterochromatin formation, has a persistent impact; a small but measurable PEV phenotype coupled with some depletion in silencing marks HP1a and H3K9me2 is observed in the adult, even when the developing zygote has two wild type alleles of *Su(var)205*. As anticipated, the HP1a mutation also shows an impact on later heterochromatin assembly (required after each mitosis), demonstrating an active role in heterochromatin formation and/or maintenance during later development. While the maternal effects *per se* are small, one consistently observes greater loss of silencing in the M+Z flies compared to the Z flies, arguing that a deficit during heterochromatin formation cannot be entirely overcome by supplying the required protein later during development.

### 2. Piwi's impact on the chromatin of adult non-gonadal somatic tissues reflects its function in heterochromatin establishment in the early embryo

**Figure 2:**
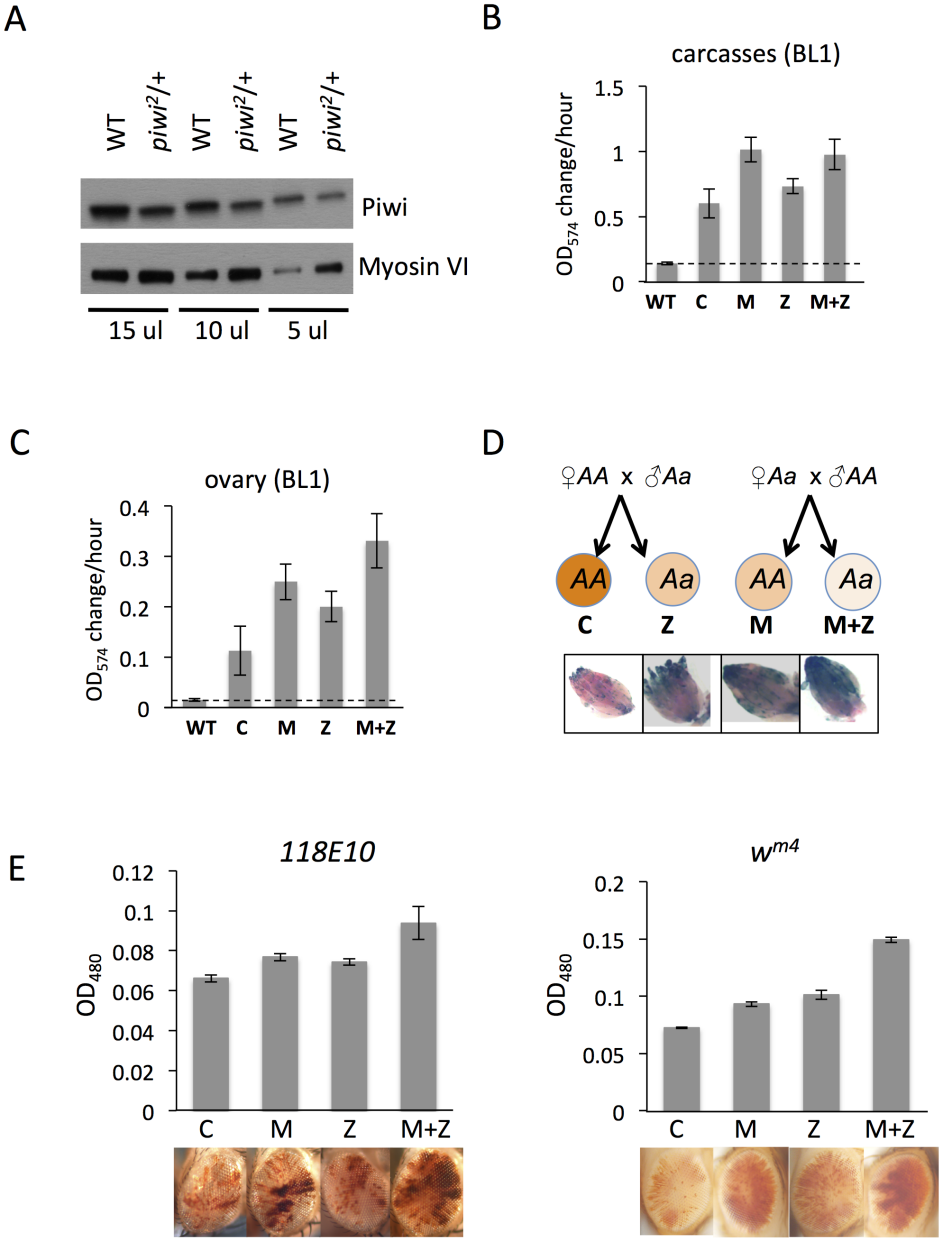
The suppression of variegation in response to Piwi depletion reflects the reduced level of Piwi protein in the early embryo. (A) Western blot analysis of Piwi proteins shows that *piwi^2^*/+ heterozygous female fly ovaries exhibit half the amount of Piwi protein found in wild type (WT). Myosin VI is used as the loading control; the volume of lysate loaded is indicated beneath. (B, C) Quantitative β-galactosidase assays show that decreased maternal loading of Piwi leads to suppression of variegation at the BL1 reporter in both non-gonadal (B) and gonadal (C) cells in adults. (D) X-gal staining in ovaries shows that both maternal and zygotic depletion of Piwi leads to elevated expression of β-galactosidase in ovaries. Depletion strategies are shown in (D). (E) Similarly, a small but consistent loss in silencing is observed on maternal or zygotic depletion of Piwi using an eye phenotype for assessment. *piwi* mutant alleles used: *w; piwi^2^/CyO* for B, C, D and the left panel of E; *w; piwi^1^/CyO* for the right panel of E. Error bars denote SEM.

It is of interest to examine the roles of both heterochromatin-specific chromosomal proteins and of the components of the RNAi system, given the potential of the latter to target assembly of the former. Piwi, the piRNA binding protein, carries small RNAs into the nucleus and is reported to be an HP1a-interacting protein; thus it has been suggested to be essential for the accumulation of local silent marks to silence targets transcriptionally [20], [23]. To ask whether Piwi functions in early embryos and/or later stages during development for the establishment of heterochromatin, we first used the *piwi^2^* null allele to reduce Piwi levels in all cell types. The ovaries of heterozygous *piwi* ^2^/+ females show a ∼2-fold decrease in levels of Piwi protein compared to wild type (Figure 2A). This presumably leads to a ∼2-fold decrease in maternal loading of Piwi into the eggs from *piwi* ^2^/+ mothers. Those offspring inheriting the *piwi* wild type allele from both parents experience maternal depletion of Piwi only (denoted as M in Figure 2), while the *piwi* ^2^/+ offspring experience both maternal and zygotic depletion (denoted as M+Z, Figure 2). Piwi is expressed primarily in gonads in adults. To see whether maternal depletion of Piwi has any effect on silencing in the non-gonadal somatic cells, we assayed the β-galactosidase activity in carcasses and ovaries separately. In M and M+Z adult flies, where Piwi is depleted in early embryos by a maternal effect, the *hsp70*-*lacZ* PEV reporter located at the 3L pericentric heterochromatin (reporter BL1) shows increased expression in carcasses relative to the relevant controls (C and Z) (Figure 2B). This demonstrates that maternal depletion of Piwi leads to less silencing of the BL1 reporter expression in non-gonadal somatic tissues assayed in adults. In ovaries, while the expression levels of β-galactosidase from the BL1 reporter in M and M+Z flies again are higher than the relevant controls C and Z, we observe that the M and Z levels are comparable, higher than that in C flies and lower than that in M+Z flies (Figure 2C). This result is also clearly shown by X-gal staining of the ovaries (Figure 2D). This pattern suggests that Piwi is important in both embryos and developing female gonadal cells for determining the silent state of the reporter. This early effect in gonadal somatic cells is in agreement with work demonstrating that maternal piRNAs are required to silence retrotransposons in ovarian somatic cells [42]. The zygotic effect observed in gonads is in congruence with the observations that Piwi is required in OSC (a cell line derived from ovarian somatic cells) and follicle cells to maintain the transcriptional silencing of transposable elements [21], [43], [44].

To further study Piwi's role in heterochromatin silencing in non-gonadal somatic cells, we used the *118E10* and *w^m4^* reporter lines to determine whether maternal depletion of Piwi has any effect in the eye lineages. We consistently observe a weak but significant increase in eye pigment levels in M and M+Z flies compared to control C and Z flies, indicating a maternal effect (Figure 2E). It is interesting that genetically wild type female flies subjected to Piwi depletion as early embryos exhibit a loss of silencing of the PEV reporter in both somatic and gonadal tissues (M in Figures 2B–E), indicating that the impact of the maternal material (proteins and RNAs) involved in heterochromatin formation is transmitted to the pole cells as well as somatic cells of the offspring at the early embryo stage [45]. Most likely this impact occurs during the initiation of heterochromatin assembly in early (1–3 hr) zygotes, a stage when Piwi is enriched in the whole embryo, including both somatic cells and germ line cells [46].

### 3. Knock-down of Piwi in early embryos has a long-lasting effect seen in late stage animals

**Figure 3:**
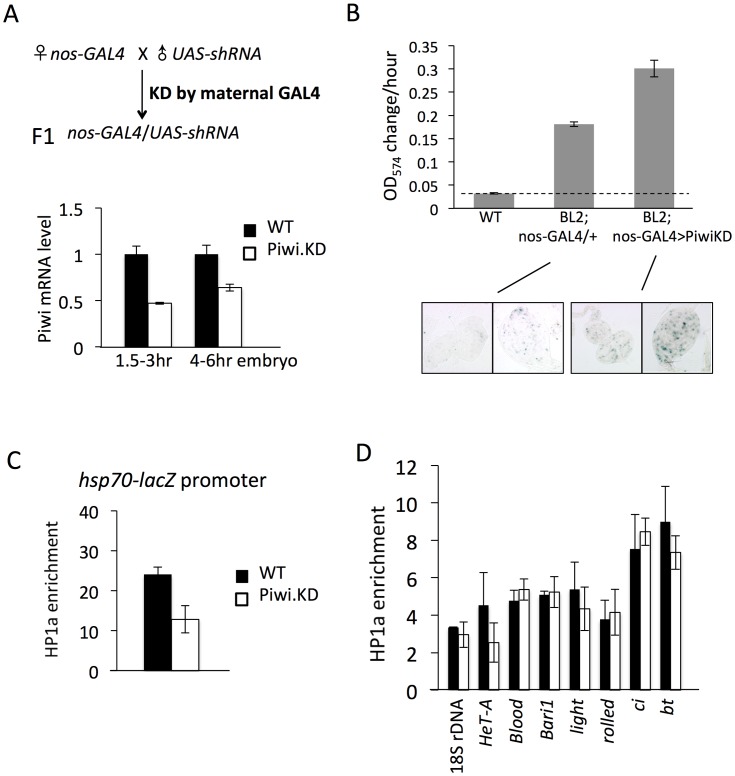
Knockdown (KD) of Piwi in early embryos has a down-stream effect on chromatin structure of a PEV reporter in late stage animals. (A) Strategies for knocking down the Piwi mRNA in early embryos. By crossing females with the germ line specific GAL4 driver (*nos*-GAL4; NGT) to males with the UAS-shRNA hairpin, Piwi mRNA is depleted in the F1 generation embryo, presumably degraded by the small RNA produced by the paternally-derived shRNA hairpin driven by maternally loaded GAL4. Expression levels are given relative to the RPL32 locus. (B) A quantitative β-galactosidase assay of whole adult males and an X-gal staining assay for β-galactosidase expression in larval imaginal discs both demonstrate that embryonic depletion of Piwi leads to the suppression of variegation of the *hsp70*-*lacZ* PEV reporter on the Y chromosome (BL2) in subsequent developmental stages. (C) ChIP-quantitative PCR analysis for HP1a enrichment levels at the promoter region of the BL2 reporter in adult males depleted for Piwi in the early embryo. (D) HP1a enrichment levels at other heterochromatic loci in adult males depleted for Piwi in the early embryo. In (C) and (D), ChIP-qPCR was performed using male adult whole flies from mothers having the NGT driver and fathers having the shRNA against Piwi. The enrichment levels are normalized to the α-actinin locus. Error bars denote SEM.

To confirm that Piwi has an early effect on heterochromatin establishment, we applied a recently developed approach to knock down (KD) its expression in the early zygote by using the female germline-specific *nanos-GAL4-tubulin* (NGT) drivers [47] with the UAS-shRNA transgenic lines produced by the TRiP project [48], [49] (Figure 3A). The efficacy of this strategy has been assessed by measuring RNA and protein levels of the KD target gene. When females with NGT drivers are crossed to males with an shRNA hairpin targeting Piwi, Piwi mRNA is observed to decrease by 2 fold in 1.5–3 hour F1 embryos, presumably as a consequence of the maternally loaded GAL4 driving the paternally provided shRNA hairpin (Figure 3A). The level of Piwi protein is decreased by ∼2-fold in these 1–2 h embryos (Figure S3).

We used the PEV reporter *hsp70*-*lacZ* located on the Y chromosome (BL2, a translocation of the transgenic reporter from the 3L tip to the Y chromosome, which shows expression in various somatic cells as well as in gonads [41]) to assay whether knocking down Piwi in the early zygote will lead to any perturbation in heterochromatin structure in later stage animals. Indeed, early zygotic depletion of Piwi (using the KD strategy in Figure 3A and analyzing the F1 progeny) leads to suppression of variegation of *hsp70*-*lacZ* in both larvae and adult animals. The expression level of *hsp70*-*lacZ*, measured by assaying β-galactosidase activity, was elevated by 2-fold in the whole animal (Figure 3B, male adults, and Figure S4, male larvae). As the NGT driver is highly specific, being expressed in female germ line and silent in male gonads (Figure S5, tested by the UAS-mCD8::GFP construct [50]), this change must be attributed to events in the early embryo. In addition, the β-galactosidase activity was assayed in larval imaginal discs using X-gal staining; this assay clearly shows that the number of cells with active *lacZ* expression has been increased following Piwi KD (Figure 3B, bottom panel).

The results obtained using the β-galactosidase assay to measure the expression levels of *hsp70*-*lacZ* demonstrate that Piwi KD in early zygotes, where heterochromatin formation is initiated, leads to suppression of variegation of this PEV reporter. These findings argue for a role for Piwi in heterochromatin establishment, with the consequences maintained through subsequent mitotic inheritance of this chromatin state during development. To test whether this occurs through a chromatin-based mechanism, we assayed the HP1a levels in the *hsp70*-*lacZ* promoter region in adult animals by ChIP-qPCR. The ChIP-qPCR result shows that the HP1a level at the *hsp70*-*lacZ* promoter region is decreased by about 2 fold in adults following KD of Piwi in early zygotes (Figure 3C). Thus, the suppression of PEV caused by the depletion of Piwi in the early zygote is a chromatin-based mechanism, in that the increased reporter expression is associated with perturbation of HP1a levels at this site.

HP1a levels were tested at other heterochromatic loci to see whether there is a widespread change in heterochromatic regions. Previous work examining the impact of Piwi depletion (or depletion of another piRNA component, spn-E) in female germ line nuclei has shown that Piwi plays a role in transcriptional silencing in the germ line for some (but not all) transposable elements (TEs), including *HeT-A, bari* and *blood*, by an HP1a-dependent mechanism [20], [23], [51]. Assaying HP1a enrichment by ChIP-qPCR, we observe that only *HeT-A* shows a 2-fold decrease at the promoter region on depletion in the early embryo (Figure 3D), while levels of HP1a associated with *blood* and *bari* appear unchanged. In addition, neither classic heterochromatin genes *light* and *rolled*, nor chromosome 4 genes *ci* and *bt*, show a significant alteration in HP1a levels (Figure 3D). This suggests that PEV reporters, at the border of heterochromatin and euchromatin, are particular targets of the mechanism involved and/or particularly sensitive reporters, and that Piwi depletion in early zygotes at this level does not lead to a dramatic impact on the heterochromatin structure as a whole. This result should be anticipated given the survival of the mutant animals.

### 4. Eye lineage-specific KD of HP1a or EGG, but not of RNAi components Piwi or AGO2, suppresses variegation

**Figure 4:**
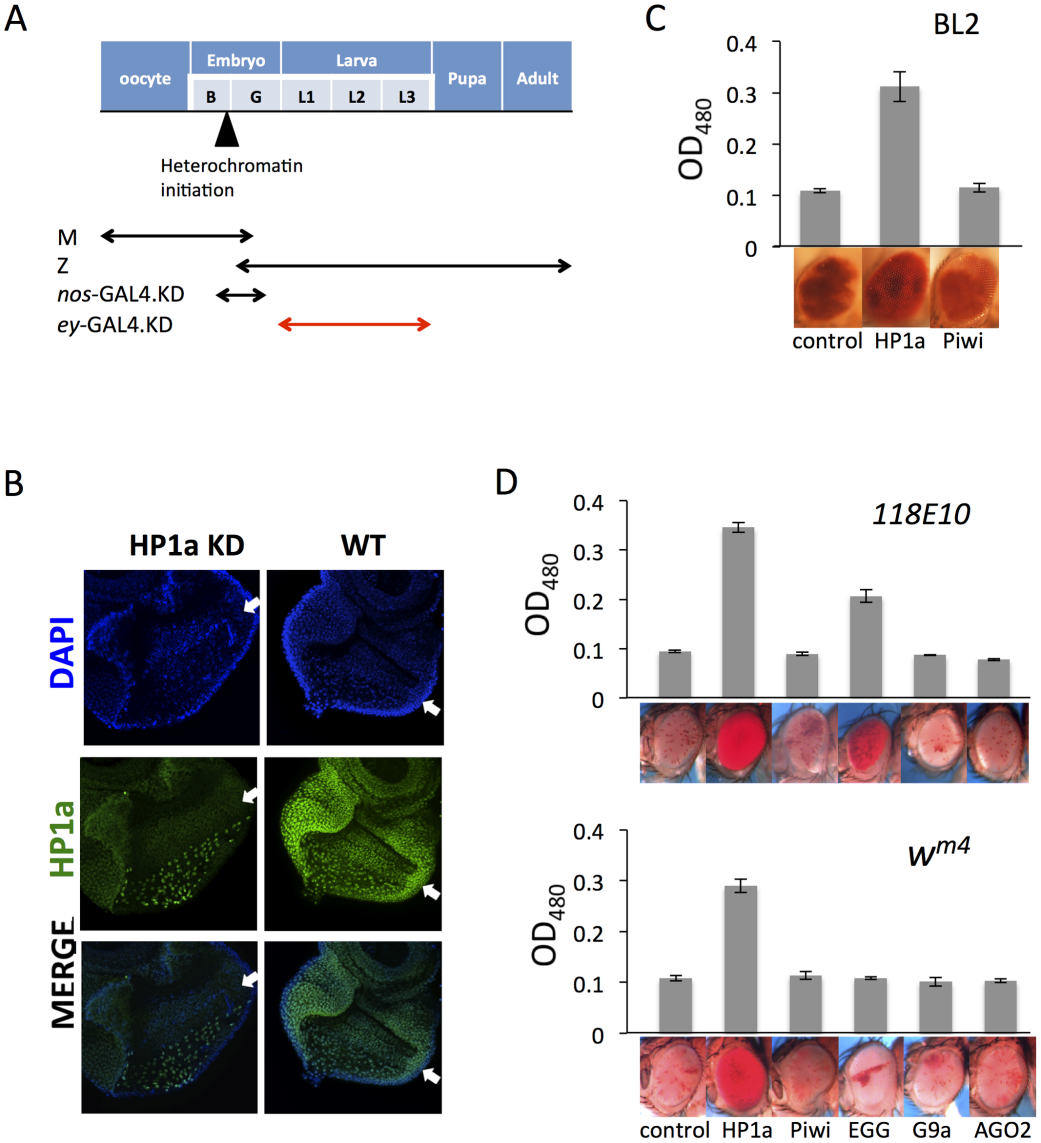
Eye lineage-specific knockdown (KD) of HP1a or EGG, but not of RNAi components Piwi or AGO2, suppresses variegation of a *white* PEV reporter. (A) Schematic illustration of the developmental stage of depletion of the protein of interest by null alleles (used in Figures 1 and 2), by the *nos*-GAL4 driver (used in Figure 3), and by the *ey*-GAL4 driver (used here). (B) Immunofluorescent staining of HP1a in the eye disc. HP1a expression is knocked down in the eye lineage using the *ey*-GAL4 driver, and recovered just behind the morphogenetic furrow where *ey*-GAL4 is shut off. Arrows point to the morphogenetic furrow. (C) Pigment assays show that depletion of HP1a by the *ey*-GAL4 driver leads to suppression of variegation of the *white* gene on the BL2 reporter, while depletion of Piwi does not. Data from adult males. (D) Pigment assay results show that depletion of HP1a or EGG, but not Piwi, in the eye lineage from *ey*-GAL4-driven KD leads to increased expression levels of *w^m4^* and of *hsp70*-*w* from the *118E10* reporter. Error bars denote SEM. Data from adult females.

To contrast the roles of those proteins involved in the maintenance of heterochromatin in somatic cells with those not required, we examined the impact of KD of different genes specifically in the eye lineage by using the eye-specific *ey*-GAL4 driver and corresponding hairpins, assaying the effects on various PEV *white* reporters. The *ey*-GAL4 transgene, under the control of the *eyeless* promoter, is active in the developing eye disc from late embryogenesis until shut off before the last cell division, following the progression of the morphogenetic furrow (MF) across the eye disc in third instar larvae ([52]; Figure 4A). The inducible feature of this method allows us to manipulate the expression levels of those genes of interest by controlling the timing in a specific tissue, as shown by others (e.g. [53], [54]). The efficacy of the *ey*-GAL4 driver in specifically knocking down HP1a expression in eye lineage cells is demonstrated by a cytological assay using the eye disc. HP1a staining is very faint before the morphogenetic furrow (where *ey*-GAL4 is active), but becomes much stronger after the morphogenetic furrow (where *ey*-GAL4 is shut off) (Figure 4B). The foci of HP1a dense regions, seen immediately after HP1a expression is restored, overlap with the DAPI-dense regions of the nuclei. The dramatic contrast in HP1a staining on either side of the morphogenetic furrow (Figure 4B, middle panel, left) demonstrates that HP1a KD by the *ey*-GAL4 driver during the eye development is very efficient.

The BL2 reporter sequence also carries the *white* gene down stream of its minimal endogenous promoter sequences [41]; the adjacent *hsp70-lacZ* and *white* reporters show concordant expression in the fly eyes [38], [41]. The eye pigment levels from the BL2 reporter were assayed to investigate the effect of HP1a or Piwi knock down in the developing eye lineage (using *ey*-GAL4) on silencing. Indeed, HP1a depletion leads to increased expression of the *white* reporter gene in eyes, while Piwi depletion has no impact (Figure 4C). In addition, pigment assays indicate that KD of HP1a results in a dramatic suppression of variegation for both *w^m4^* and the *hsp70-w* reporter line *118E10*, while KD of Piwi and AGO2 in these somatic cells does not have any impact on the reporters (Figure 4D). The KD of EGG, which codes for an H3K9 histone methyl transferase, results in different impacts depending on the location of the reporters, as anticipated. For the reporter on chromosome 4 (*118E10*), EGG KD in eye-lineage cells results in significantly increased *hsp70-w* expression (suppression of PEV), while for the reporter juxtaposed to the pericentric heterochromatin of the X chromosome (*w^m4^*) there is less effect. In the latter case, while the quantitative pigment assay does not show a significant difference, the pattern of the pigment in the eye does change, exhibiting a non-uniform distribution instead of a uniform “pepper and salt” pattern (Figure 4D). A specific role for EGG in maintaining the heterochromatic nature of chromosome four has been previously demonstrated [54]–[57]. No impact was seen on KD of G9a, as anticipated. The results above argue that HP1a and EGG, but not RNAi components Piwi or AGO2, are actively involved in the maintenance of heterochromatin at reporter sites in dividing somatic cells during development after the late embryo stage.

### 5. Piwi null mutant animals exhibit only small changes in bulk HP1a distribution

The PEV assays shown above argue that for somatic tissues in flies, Piwi's impact on heterochromatin formation stems from its function in the early embryo, a stage at which Piwi is enriched in both pole cells and the bulk of the embryo [46]. After the onset of zygotic transcription, Piwi expression is found primarily in the gonads. The X-gal staining and quantitative β-galactosidase assays (Figures 2–3) suggest that Piwi depletion in early embryos contributes to an altered chromatin state seen in later stage animals. As the majority of the larval tissues are somatic, we used the larval stage to investigate the impact of embryonic depletion of Piwi on the HP1a enrichment profile. Data were obtained from *piwi^2^*/*piwi^2^* null larvae (offspring of *piwi^2^*/+ heterozygous parents), which have ∼50% maternally loaded Piwi protein compared to wild type in early embryos (Figure 2A). An HP1a ChIP-array assay was performed to study the genome-wide impact of early zygotic Piwi depletion on chromatin structure.

**Figure 5:**
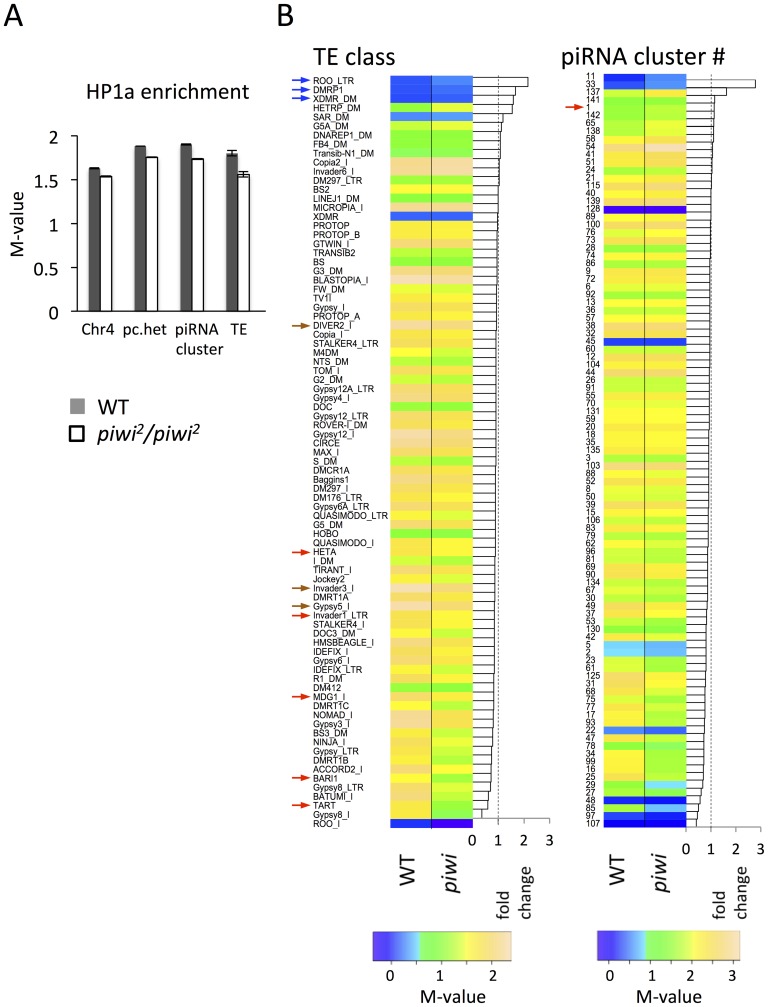
Lower HP1a enrichment at repetitious sequences in *piwi^2^/piwi^2^* mutant larvae. (A) HP1a enrichment in heterochromatin regions is slightly lower in *piwi^2^/piwi^2^* mutant larvae than in wild type. This is most apparent for TEs. WT  =  wild type; pc.het  =  pericentric heterochromatin; TE  =  transposable elements. (B) HP1a enrichment profile for various TE classes and piRNA clusters. The arrows point to the classes of TEs and piRNA clusters mentioned in the text.

In *piwi^2^/piwi^2^* null larvae (50% depletion of Piwi in the early embryo), HP1a enrichment (measured by M value, see Methods for details) exhibits a small decrease in all heterochromatic regions investigated: pericentric heterochromatin (6.7% decrease), chromosome 4 (5.7%), piRNA clusters (defined by [58]) (8.7%). The largest decrease was observed for TEs (13.4%) (Figure 5A). We further calculated the HP1a enrichment levels for individual TE classes to look for any differences. In wild type larvae, HP1a enrichment varies among different TE classes, ranging from very little HP1a enrichment (e.g. *roo, DMRP1* and *XDMR*; blue arrows, Figure 5B), to M-values larger than 2 (*gypsy5*, *invader3*, *DIVER2*; brown arrows, Figure 5B) (see Methods for detailed definition of the M-value). In *piwi^2^/piwi^2^* null larvae, the HP1a levels for most TE classes were decreased (69 out of 83 classes investigated, Figure 5B); this was found for *Bari1, Invader1, mdg1* and telomere associated *Het-A* (red arrows, Figure 5B), TEs that are sensitive to Piwi depletion in the germ line [20], [23], [51]. A second telomere associated non-LTR element *TART* also falls into the group with the largest HP1a reduction. However, HP1a levels do not change much for some TE classes, including *roo*, where HP1a enrichment is very low and does not change on Piwi depletion in the female germ line (see also [20]). Consistent with this observation, we find that depletion of HP1a in parents and developing animals (*Su(var)205^04^/Su(var)205^05^* larvae from heterozygous parents, data from [57]) results in loss of the heterochromatin mark H3K9me2 at TE classes such as *HET-A* and *TART*, but not at *roo* (Figure S6), suggesting that different TEs have different sensitivity to the Piwi-HP1a silencing system. These observations indicate that HP1a binding at TEs, and the impact of the piRNA system on that binding, varies; the pattern of association seen here mimics that reported in earlier studies of Piwi function in TE silencing in the ovary [20], [23]

Similarly, the HP1a enrichment in piRNA clusters shows an overall decrease in *piwi^2^/piwi^2^* mutant larvae, but the changes differ in different clusters (Figure 5B). Some piRNA clusters, including the longest piRNA cluster in the 42AB region (cluster #1 defined by [58]; red arrow in Figure 5B right), actually show an increase in HP1a enrichment, consistent with the previous report for this region [59]. But for the majority of the piRNA clusters (80 out of 96 investigated, Figure 5B, right), HP1a enrichment decreases (generally to a small extent) with Piwi depletion.

Piwi is reported to be essential for the recruitment of active histone marks in the sub-telomeric 3R-TAS region, with loss of Piwi leading to the increase of HP1a and silencing of *white* reporters inserted in 3R-TAS in flies [60]. There are few probes in the array that could be uniquely mapped back to the highly repetitious TAS region, so we plot the HP1a enrichment at the tips of the chromosome arms 2L, 2R, 3L and 3R to study the role of Piwi in the recruitment of HP1a in sub-telomeric regions. Indeed, increased HP1a enrichment is observed in the most distal regions of the assembled chromosome sequences (Figure S7) in *piwi^2^/piwi^2^* null larvae, suggesting an active role of Piwi in those sub-telomeric regions.

In *piwi^2^/piwi^2^* null animals, the gonads are tiny and rudimentary. One might wonder whether the small changes in HP1a association with TEs could result from the absence of gonadal tissues in *piwi^2^/piwi^2^* null larvae. To consider this question we analyzed the HP1a levels in ovary. The HP1a enrichment for the individual TE classes is highly correlated between ovary and larvae (Figure S8A, R^2^ = 0.874). If the reduced HP1a level seen for some TEs in *piwi^2^/piwi^2^* null larvae were a consequence of the depletion of gonadal tissue, one would expect to see that those TEs would have higher HP1a enrichment in ovary compared to larvae (should fall below the dashed line); this is not observed when the 10 TEs showing the largest HP1a reduction in *piwi^2^/piwi^2^* null larvae are plotted (red dots in Figure S8A). Furthermore, there is no correlation between the HP1a reduction in *piwi^2^/piwi^2^* null larvae and the HP1a levels in ovary (Figure S8B). Thus, the observed HP1a change is unlikely to be due to the depletion of gonadal cells in the mutant animals. Reduced HP1a levels in pericentric heterochromatin and TEs have also been observed in newly eclosed *piwi* null adult flies, which have little gonadal tissue [61].

Overall, the HP1a profiles demonstrate that depletion of Piwi results in a small overall reduction in HP1a at heterochromatic sequences in general, with variation among different heterochromatin classes and elements. Among these, HP1a enrichment drops most significantly at TEs on Piwi depletion. This result argues for a multiplicity of mechanisms for heterochromatin formation, with Piwi playing a significant role at a subset of TEs.

## Discussion

**Figure 6:**
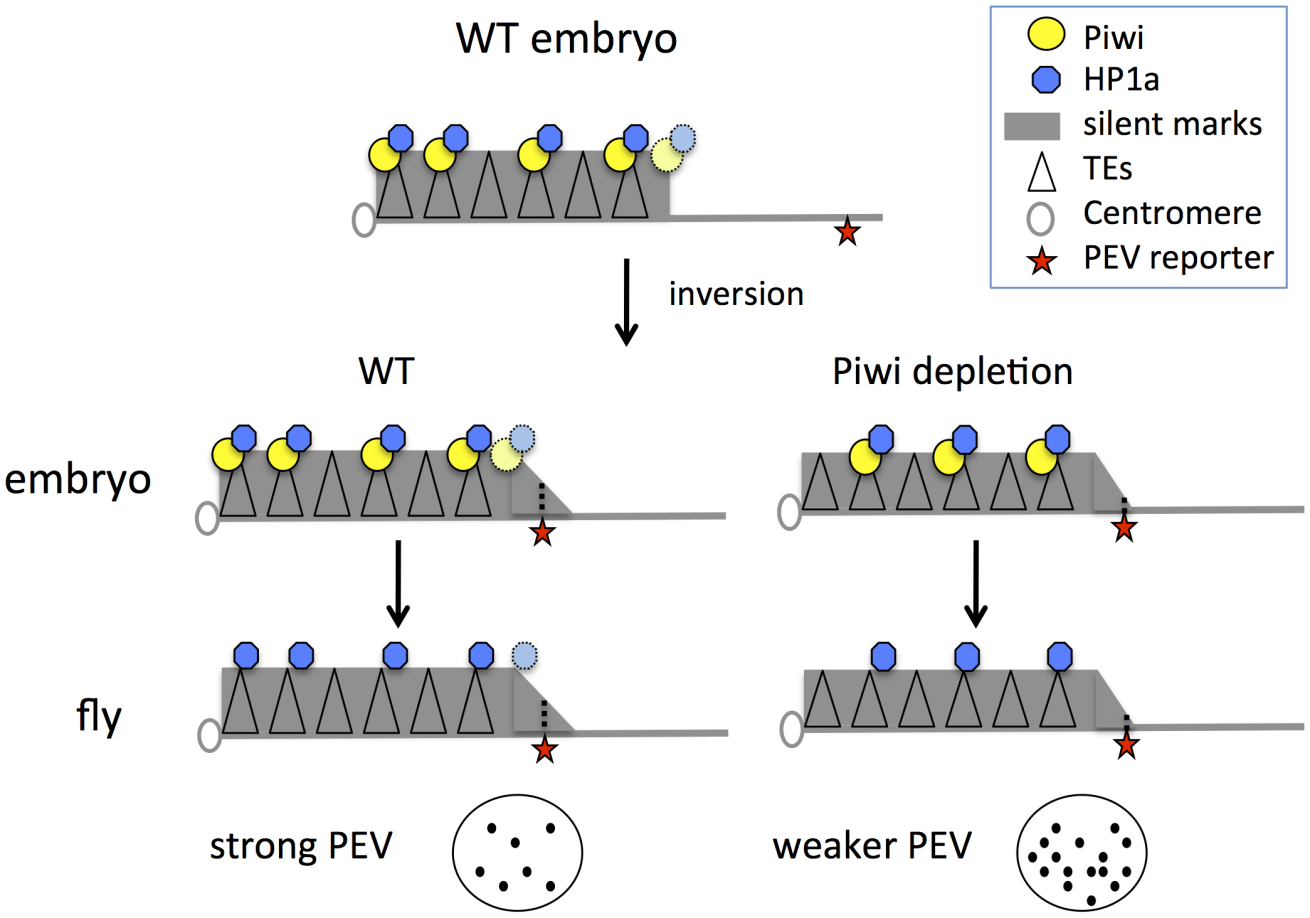
Model for Piwi's role in the heterochromatin formation and PEV reporter silencing. In wild type embryos, Piwi guides HP1a deposition to a subset of TEs in heterochromatin, maintaining normal levels of silent marks. PEV reporter silencing is dependent on the spreading of heterochromatin components from the heterochromatin mass. During development, the silent state is maintained during mitosis independent of Piwi. When Piwi is depleted at the critical stage of heterochromatin establishment in the embryos, the HP1a level in heterochromatin in this region is lower; the reduced HP1a enrichment results in loss of silencing of the sensitive PEV reporter, a state that is propagated to adult animals. Depletion of HP1a in the early embryo has a similar effect, even when HP1a is present at normal levels during mitotic cell divisions. Piwi may act at the border regions between euchromatin and heterochromatin in particular, to prevent aberrant transcription, making PEV reporters particularly sensitive to the depletion of Piwi.

These results, coupled with earlier findings, support a model for heterochromatin targeting that utilizes Piwi in the early zygote (Figure 6): we suggest that Piwi and the associated piRNA system are required (directly or indirectly) to guide HP1a to a subset of TEs, and that the deposition of HP1a further recruits other components to establish H3K9me2-enriched heterochromatin status in those TE regions. Specificity could be achieved via a base-pairing mechanism utilizing piRNAs [19]. Subsequent mitotic transmission of this HP1a/H3K9me2 enriched heterochromatic state during development does not appear to depend on the piRNA system. This targeting mechanism may be of primary importance for TEs in border regions between heterochromatin masses and adjacent euchromatin, the situation for PEV reporters utilized here. When Piwi is depleted, the HP1a level is significantly decreased at these sites. Some loss of HP1a is seen in general in heterochromatic regions, presumably because heterochromatin is enriched in TEs and other repetitious elements. Thus the silencing of PEV reporters, which are dependent on the spreading of the local heterochromatin, can be released. The silent chromatin state is apparently transmitted by the heterochromatin system during development, when the piRNA system is largely absent in non-gonadal somatic cells.

Using mutant alleles, we assayed the effect of maternal depletion (which results in depletion in early embryos) and zygotic depletion of HP1a or Piwi on the expression of PEV reporters. Functional HP1a depletion in either the early zygote or developing animals leads to suppression of variegation of the PEV reporters, coupled with decreased levels of HP1a itself as well as the silencing mark H3K9me2 in the reporter regions (Figure 1). This suggests a critical role for HP1a in both early establishment and subsequent maintenance of heterochromatin, and demonstrates that the impact of early depletion can be seen using an adult phenotype, even when wild type alleles of HP1a are present in the developing zygote. In the case of Piwi, only maternal or early zygotic depletion has a significant effect on the reporters in non-gonadal somatic cells (Figures 2–4). Surprisingly, zygotic Piwi depletion in embryos from wild type mothers does cause a small decrease in PEV silencing of the BL1 reporter in carcasses (Figure 2B), and of *118E10* and *w^m4^* reporters in eyes (Figure 2E). However, this effect is not as significant as that caused by maternal depletion. A small zygotic effect of Piwi depletion is consistent with prior observations [62]. At the same time, the results of Piwi knock down in the eye lineage argue that Piwi is dispensable for the maintenance of heterochromatin silencing after embryogenesis (Figures 4C, 4D). Note that the *ey*-GAL4 driver becomes active in late embryogenesis, much later than the onset of zygotic expression in the 2-hour embryo.

Overall, the data demonstrate that Piwi's role in recruiting HP1a and other components to some TE regions happens early in development, while HP1a is essential for heterochromatin formation during every cell cycle. This is in congruence with their expression patterns. Piwi mRNA is present in gonadal cells and early embryos, with little detectable expression in non-gonadal somatic cells, with some exceptions (e.g., larval fat body, possibly nerve cells), while HP1a is expressed in all cells/tissues during development [40], [63]. Thus any HP1a-Piwi interaction likely occurs in gonadal cells and in early embryos, where they are both highly enriched and observed to be nuclear proteins. These stages are also enriched in small RNAs and piRNA pathway components [64], supporting a model of piRNA-mediated heterochromatin assembly. As it is a structural protein of heterochromatin, one would anticipate that HP1a would be essential for heterochromatin formation in any dividing cell (such as those in the eye imaginal disc) when heterochromatin is re-established after DNA replication as is observed (Figure 4). A second protein found to be important for silencing state maintenance for some reporters is the histone methyltransferase EGG (Figure 4D; also [54]). EGG has been suggested to be essential for heterochromatin formation in specific regions, including chromosome 4 (in somatic cells; [54]–[57], [65]) and piRNA clusters (gonads; [65]).

It is of interest that cells in the mature organism “remember” the loss of HP1a in the early zygote, exhibiting HP1a and H3K9me2 reduction in the reporter promoter region in the adult (Figure 1). The depletion of HP1a at the critical stage of heterochromatin establishment during early development, even when the overall HP1a level is presumably recovered soon after the onset of zygotic transcription, results in diminished heterochromatic regions that apparently cannot be fully re-established, and only partially recover. This implies that both genetic and environmental insults sustained at the critical embryonic stage can have a long-lasting impact on the individual.

The reporters exhibiting PEV used here either lie near the break point between heterochromatin and euchromatin caused by inversion or translocation (e.g. BL1, BL2 and *w^m4^*), or have been inserted into heterochromatic domains by P element transposition (e.g. *118E10*). Their silencing is dependent on the spreading of the adjacent heterochromatin structure, making them sensitive to even small changes in the heterochromatin environment and chromatin assembly systems [66]. For example, when Piwi is knocked down in the early embryo, we observe suppression of variegation of the BL2 reporter coupled with significant HP1a loss at the promoter of the reporter (Figure 3). However, no dramatic change of HP1a enrichment in is observed in most other heterochromatic regions (Figure 3). The sensitivity of the BL2 reporter to Piwi depletion might be explained by its position at the edge of a heterochromatic mass, and the requirement for spreading of the heterochromatic assembly. The HP1a ChIP-array data in *piwi* mutant larvae further confirms that depletion of Piwi will lead to a small decrease in the HP1a level at some TE classes, coupled with an overall small decrease of HP1a levels in heterochromatic sequences (Figure 5). However, the data obtained from the ChIP-array includes only the unique probes in the assembled genome sequence, so only a small portion of the TEs have been analyzed. It is possible that the actual overall decrease of HP1a enrichment is greater, as most of the TE sequences are not included in this analysis. Nonetheless, the PEV reporters may be particularly sensitive to Piwi manipulation, either because of their dependency on spreading of heterochromatin, or because the Piwi-dependent response itself is triggered by transcription, more likely to occur in these flanking regions.

While our studies have focused on the role of Piwi, the resulting model is consistent with earlier work examining several components of the piRNA pathway (Piwi, Aubergine, Armitage, Spn-E). Mutations in these components are reported to have an impact on the repression of transcription and maintenance of a closed chromatin structure for several TE classes when assayed in the female germ line [20], [23], [51]. We demonstrate here two additional features: first, that maternal depletion of Piwi has an impact on silencing PEV reporters that can be seen in somatic cells of larvae and adults, and second, that depleting Piwi in early zygotic cells (but not maternally) also impacts PEV assayed in later stages.

Our results further suggest that the piRNA system observed here most likely acts in the context of multiple mechanisms for heterochromatin formation. In the yeast *S. pombe* the RNAi system is redundant with other heterochromatin protein interaction systems in heterochromatin establishment [12]; such DNA-protein interaction systems have also been inferred in *Drosophila* [67], [68]. The interplay among these systems remains to be investigated. The system of selective depletion developed here should allow further investigation of the role of various components in targeting and maintaining heterochromatin at different heterochromatin domains.

## Methods

### 1. *Drosophila* stocks

Stocks were maintained and crosses carried out using cornmeal sucrose-based medium [69]; fly culture conditions were set at 25°C with 70% humidity. Stocks used in this study are listed in Table S1.

### 2. Pigment assay

Ethanol-based pigment extraction and quantification was performed as described in Sun et al 2004 [70] with some minor modifications. Flies were homogenized in 250 µl pigment assay buffer, followed by incubation at 65°C for 10 minutes for pigment extraction. A final volume of 150 µl of pigment extract was used to read OD at 480 nm. For each assay, data from 4–8 samples (each sample made up of five 3–6 day old flies, randomly picked from the population) were collected.

### 3. X-gal staining and β-galactosidase quantitative assay

Detection of β-galactosidase in adult testes and ovaries was performed by a modification of the protocol described in Gonczy et al 1992 [71]. Flies were heat shocked at 37°C for 1 hour and allowed to recover at 25°C for 30 minutes before dissection or freezing for quantitative assays. Ovaries from 2–4 day old flies (heat shocked as described) were dissected in PBS-T (phosphate-buffered saline, 0.1% Triton) and fixed in Glutaraldehyde Fixative (2.5% glutaraldehyde, 50 mM PIPIEs) for 10 minutes. Imaginal discs from 3^rd^ instar larvae were dissected in PBS and fixed in PBS-4% formaldehyde for 15 minutes. Tissues were incubated in 0.2% X-gal staining solution at 37°C for an appropriate time to visualize staining. The mutant and wild type control samples were handled in parallel to insure equivalent staining conditions and times. For quantitative galactosidase assays, flies or tissues were homogenized in 300 ul of assay buffer (50 mM potassium phosphate, 1 mM MgCl_2_, pH 7.5), followed by spinning to pellet the debris. An aliquot of the extract was transferred to CPRG solution (1 mM Chlorophenol Red β-D-galactopyranoside in assay buffer) and the OD at 574 nm measured at intervals over a 2-hour period. The β-galactosidase activity was calculated as a function of the change in OD. For the whole animal assay, data from 4–8 samples (five 3^rd^ instar larvae or 2–4 day old flies each, randomly picked from the population) were generated. The male flies used for the assays in Figure 3 were offspring of 5-day post-eclosion (or older) mothers. For the dissected ovaries and carcasses, data from 5–8 samples (15 flies each, randomly picked from the population) were generated. The ovaries were dissected in PBS from 2–4 day old females fed with yeast.

### 4. RNA extraction and quantitative RT-PCR

RNA was isolated in TRIzol (Invitrogen) following the vendor's instructions. For each sample, tissues were homogenized in 0.5 ml TRIzol by using an electric grinder for 1–2 minutes. After DNase I treatment, RNAs were used for cDNA synthesis by using oligoA primer and the SuperScrit III kit (Invitrogen). The cDNAs were used as template for quantitative PCR to measure the abundance of a certain transcript. Quantitative PCR was performed using iQ SYBR Green Supermix (Bio-Rad) on a Cepheid Smart Cycler. Primers used are listed in Table S2. Results were analyzed by using the ΔΔCT method [72] using RPL32 as the control locus. Fifteen pairs of ovaries were collected for each RNA sample; for embryos, 200–300 embryos were used for each RNA preparation. Two biological and two technical replicates were performed and analyzed.

### 5. Immunostaining of larval discs

Imaginal discs were dissected in PBS from 3^rd^ instar larvae, and fixed in 4% formaldehyde in PBS for 15 minutes. The primary antibodies used were C1A9 anti-HP1a (1∶10) [73]. Alexa Fluor-conjugated antibodies (Invitrogen) were applied as secondary antibodies. Images were collected on a Nikon A1 confocal microscope.

### 6. Immunoblotting

Western blot analysis was performed by standard methods using whole cell lystae from staged embryos or nuclei from ovaries. Primary antibodies used were: P4D2 anti-PIWI (1∶100) [74], 3C7 anti-myosin VI (1∶20) [75], and JLA20 anti-actin (1∶100; from Developmental Studies Hybridoma Bank). Chemiluminescent detection of HRP (horseradish peroxidase) conjugated goat secondary antibodies (KPL) was performed according to vendors' instructions.

### 7. Chromatin immunoprecipitation, quantitative PCR

Chromatin preparation and chromatin immuno-precipitation were carried out following the modENCODE protocols (http://www.modencode.org) using antibodies wa191 anti-HP1a (1∶50) [76] or anti-H3K9me2 (1∶100, Abcam 1220). Homozygous *piwi* mutant third instar larvae were recovered from the stocks carrying the *piwi^2^* [77] allele over a GFP balancer by selecting for lack of GFP. For each chromatin preparation, 1 gram of third instar larvae or 2–4 day old flies (enough for 6 ChIP samples) are collected and homogenized in liquid nitrogen. Following formaldehyde fixation, nuclei are prepared and lysed, followed by sonication for 5 times 5 minutes using a Bioruptor (Diagenode). The size of the DNA fragments after sonication is about 200–500 bp. The relative enrichment of each mark at the designated region was determined by quantitative PCR (iQ SYBR Green Supermix, Bio-Rad). Primers used are listed in Table S2. The pull-down efficacy of each ChIP at each locus was determined by using input sample dilutions. Relative enrichment at a given locus was then determined by normalizing the pull-down efficacy of the target locus over α-actinin pull-down efficacy. Two biological and 2–4 technical replicates were performed and analyzed.

### 8. Array conditions and data processing

Array hybridization conditions are as previously described [5]. Two biological replicates are performed for arrays. The M value (log_2_ ratio of signal intensities between ChIP and input), data normalization and identification of regions (or peaks) with significant enrichment were performed as described [5], [57], [78]. For heatmap analyses, 500 bp bins were used to average the enrichment levels. Heterochromatin/euchromatin border positions previously identified for larvae by H3K9me2 enrichment were used to define pericentric heterochromatin [78]. The accession numbers for the array data sets are: GSE44884 (HP1 wa191.D.mel 3rd Instar Larvae Nuclei piwi2 mutant) and GSE45523 (HP1 wa191.ovary).

## Supporting Information

Figure S1 The CyO balancer does not show significant suppression or enhancement of PEV for any of the reporters in the crosses used here, BL1 (A - quantitative β-galactosidase assay), *w^m4^* (B - pigment assay) or *118E10* (C - eye photo).
(TIFF)

Figure S2 Pigment assays shows the suppression of variegation effect of maternal depletion of functional HP1a on the *hsp70-w* reporter *118E10* on chromosome 4 in male adult flies. The crosses are shown on the right.
(TIFF)

Figure S3 Western blot analysis demonstrates that Piwi protein is reduced by about 2-fold in the 1–2 h embryo lysate following KD in the early embryo by the method shown in Figure 3A. Actin is used as the loading control; the volume of lysate loaded is indicated above the figure.
(TIFF)

Figure S4 Quantitative β-galactosidase assay for *lacZ* expression demonstrates that embryonic depletion of Piwi leads to the suppression of variegation of the *hsp70*-*lacZ* PEV reporter on the Y chromosome (BL2) in 3^rd^ instar larvae. KD method is shown in Figure 3A; F1 larvae were analyzed.
(TIFF)

Figure S5 The *nos*-GAL4 driver used in the study has no impact in testis (NGT driver, Bloomington stock #32564). (A) The NGT stock is unable to drive UAS-mCD8::GFP expression in testis, while the MTD driver is able to do so (maternal triple driver, Bloomington stock #31777). The testis was dissected, fixed and examined under the microscope for fluorescent signal. (B) The Piwi signal in the testis of the NGT>shRNA-Piwi adult males does not change compared to wild type. The asterisk points to the terminal epithelium cells, which exhibit auto-fluorescence in the testis of all flies assayed. The arrow points to the germ line cells at the tip of the testis.
(TIFF)

Figure S6 H3K9me2 enrichment profile in various TE classes in *Su(var)205^04^*/*Su(var)205^05^* mutant larvae. While absolute values vary, loss of HP1a consistently leads to a loss of H3K9me2. WT = wild type; *Su(var)205^04^/Su(var)205^05^* = HP1a depleted mutant larvae.
(TIFF)

Figure S7 HP1a enrichment (M-value) in regions adjacent to the tips of the second and third chromosome arms in wild type and *piwi^2^*/*piwi^2^* mutant larvae. For each chromosome arm, the 50-kb sequence adjacent to the end of the mapped assembly is shown.
(TIFF)

Figure S8 HP1a enrichment over TEs in female adult ovary. (A) The HP1a levels over different TEs are plotted, comparing ovary and 3^rd^ instar larvae. Those TE classes showing the most HP1a reduction in *piwi^2^*/*piwi^2^* mutant larvae are plotted as red circles. In general, the HP1a enrichment seen in ovary correlates with that seen in larvae. (B) HP1a reduction over TEs in *piwi/piwi* null larvae is not correlated with the HP1a enrichment in the ovary. The HP1a fold change in *piwi/piwi* null larvae (Y axis) is compared to enrichment levels in the ovary of wild-type adult females; no correlation is observed.
(TIFF)

Table S1 Fly lines used in this study.
(DOCX)

Table S2 Primers used for quantitative PCR.
(DOCX)
