# Supplementary figures and images for: Maternal Depletion of Piwi, a Component of the RNAi System, Impacts Heterochromatin Formation in Drosophila
Source: PLoS Genet. 2013 Sep 19;9(9):e1003780. doi: 10.1371/journal.pgen.1003780 (PMC3777992; doi:10.1371/journal.pgen.1003780)

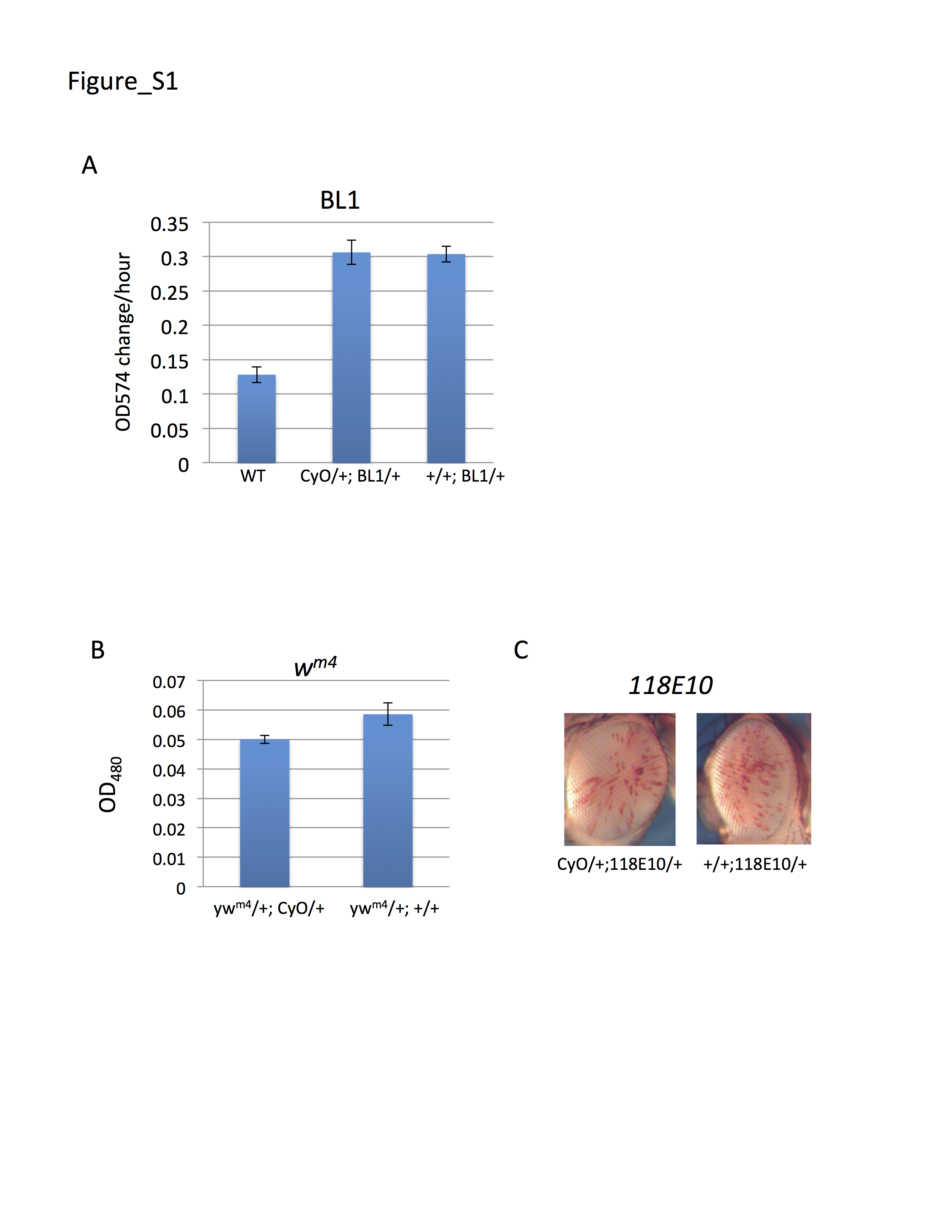

Supplement: Figure S1 — The CyO balancer does not show significant suppression or enhancement of PEV for any of the reporters in the crosses used here, BL1 (A - quantitative β-galactosidase assay), wm4 (B - pigment assay) or 118E10 (C - eye photo). (TIFF) [file pgen.1003780.s001.tiff]

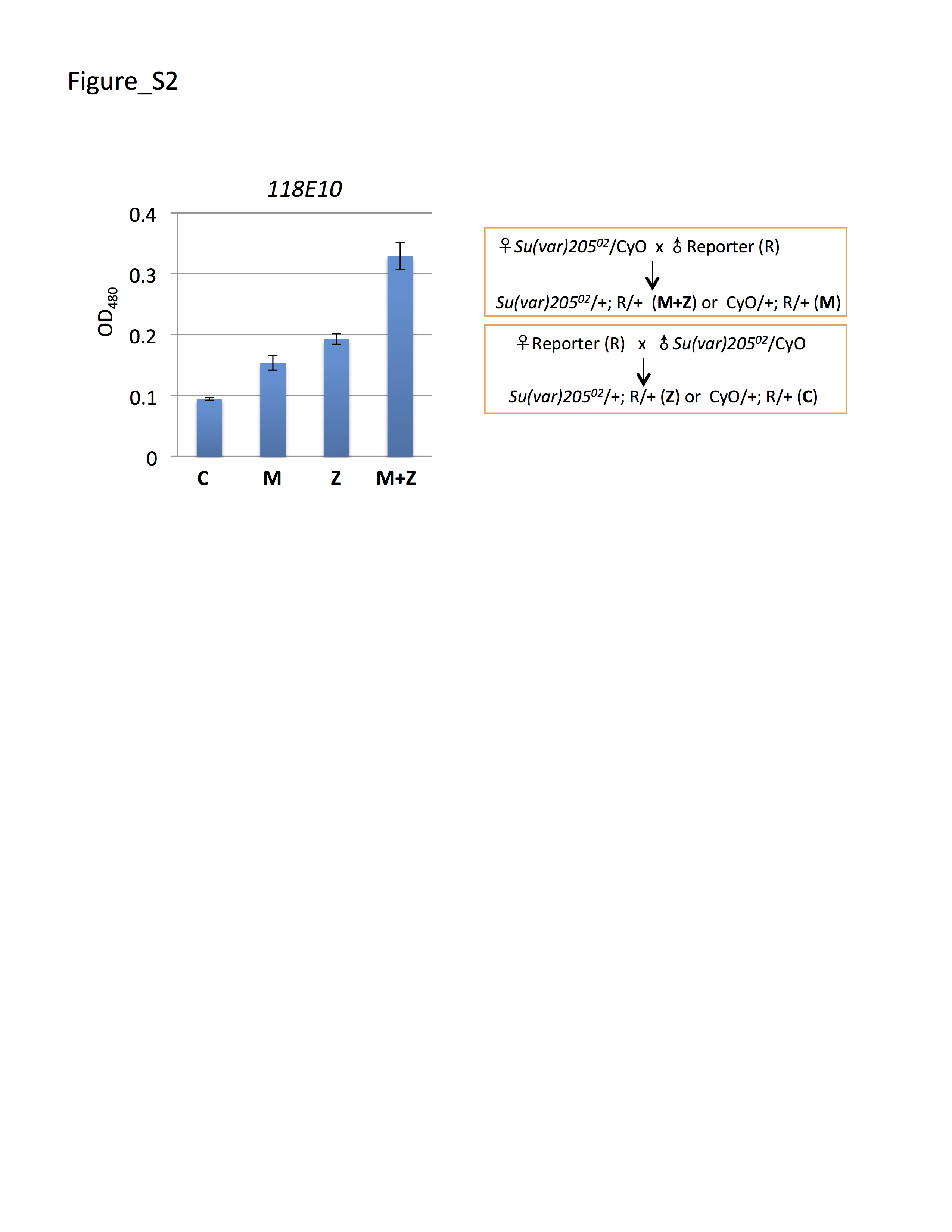

Supplement: Figure S2 — Pigment assays shows the suppression of variegation effect of maternal depletion of functional HP1a on the hsp70-w reporter 118E10 on chromosome 4 in male adult flies. The crosses are shown on the right. (TIFF) [file pgen.1003780.s002.tiff]

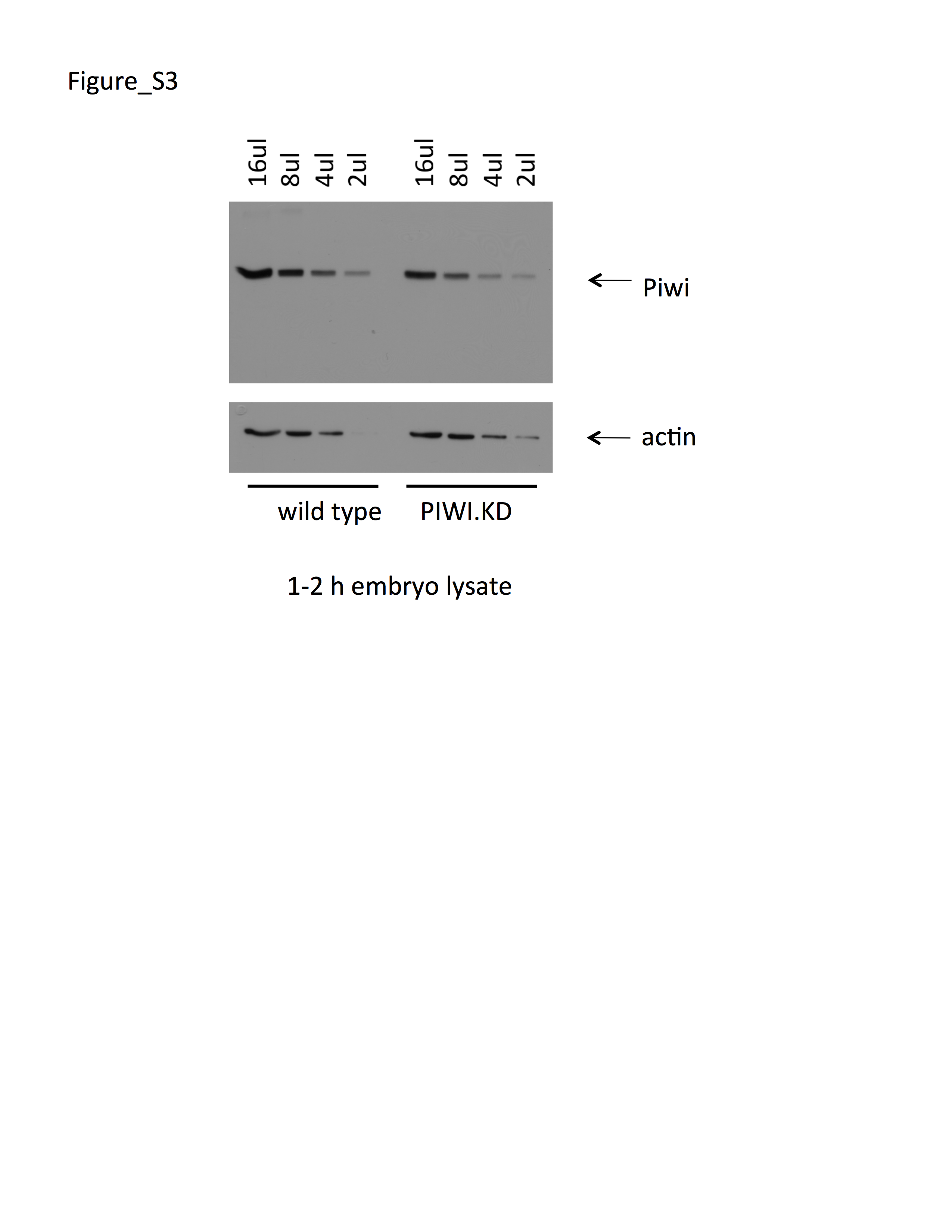

Supplement: Figure S3 — Western blot analysis demonstrates that Piwi protein is reduced by about 2-fold in the 1–2 h embryo lysate following KD in the early embryo by the method shown in Figure 3A. Actin is used as the loading control; the volume of lysate loaded is indicated above the figure. (TIFF) [file pgen.1003780.s003.tiff]

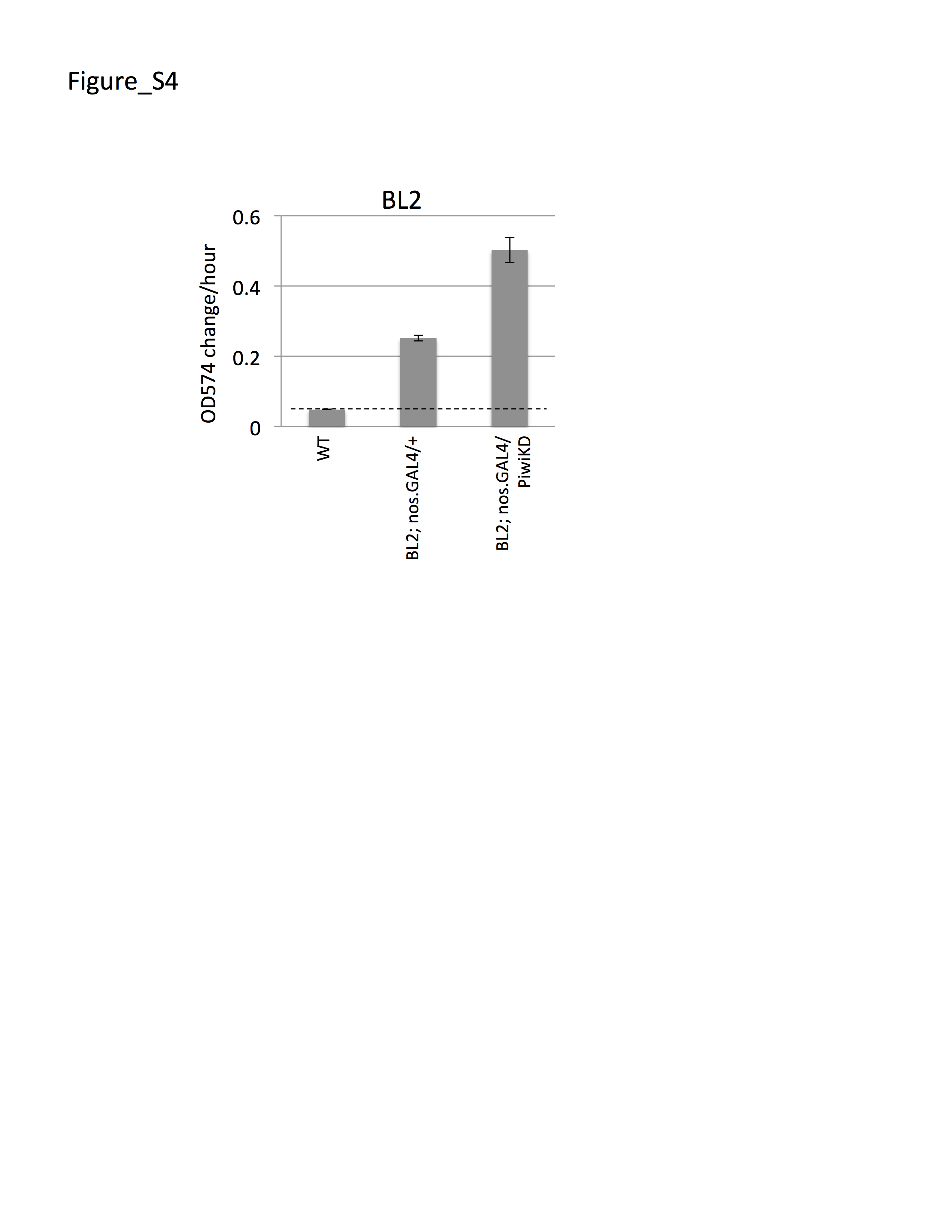

Supplement: Figure S4 — Quantitative β-galactosidase assay for lacZ expression demonstrates that embryonic depletion of Piwi leads to the suppression of variegation of the hsp70-lacZ PEV reporter on the Y chromosome (BL2) in 3rd instar larvae. KD method is shown in Figure 3A; F1 larvae were analyzed. (TIFF) [file pgen.1003780.s004.tiff]

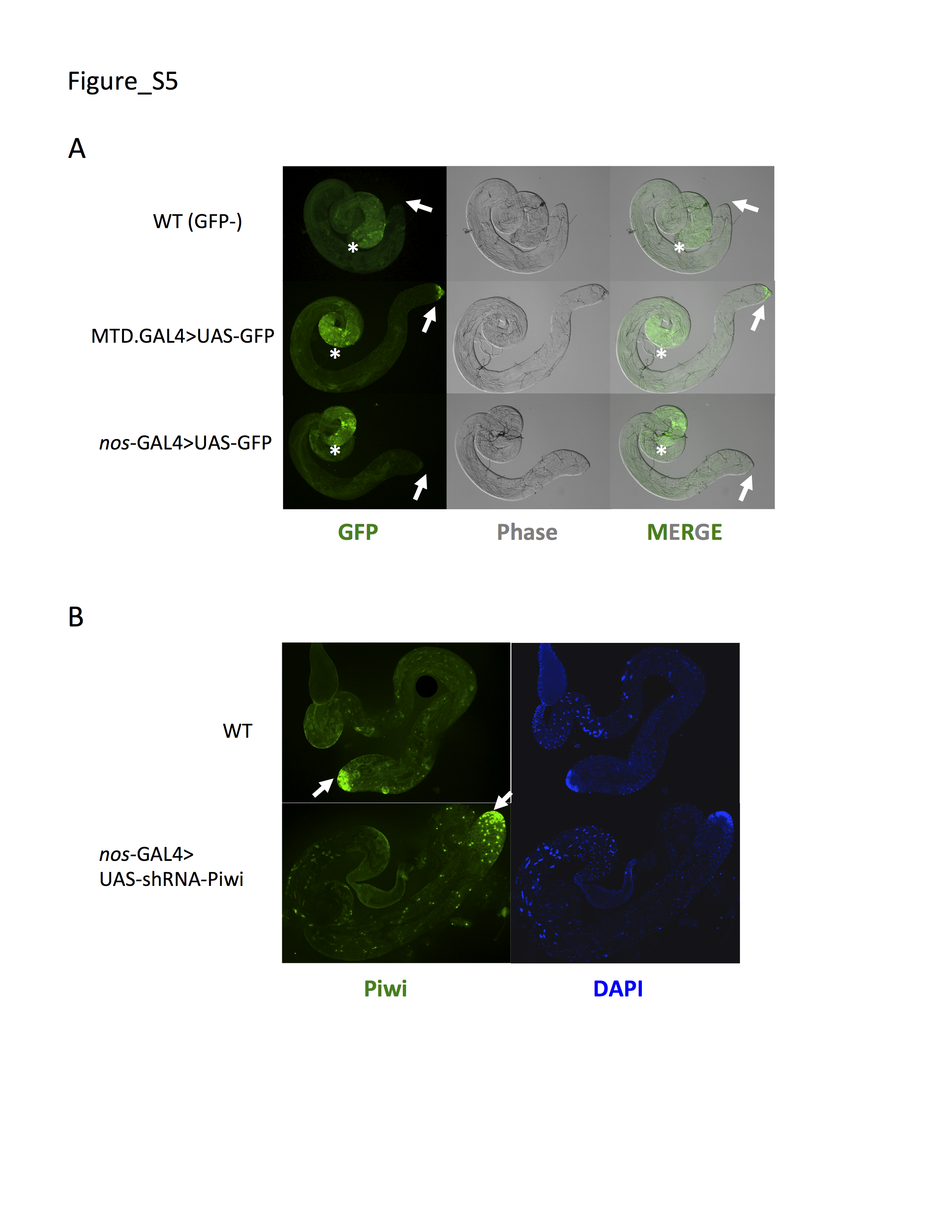

Supplement: Figure S5 — The nos-GAL4 driver used in the study has no impact in testis (NGT driver, Bloomington stock #32564). (A) The NGT stock is unable to drive UAS-mCD8::GFP expression in testis, while the MTD driver is able to do so (maternal triple driver, Bloomington stock #31777). The testis was dissected, fixed and examined under the microscope for fluorescent signal. (B) The Piwi signal in the testis of the NGT>shRNA-Piwi adult males does not change compared to wild type. The asterisk points to the terminal epithelium cells, which exhibit auto-fluorescence in the testis of all flies assayed. The arrow points to the germ line cells at the tip of the testis. (TIFF) [file pgen.1003780.s005.tiff]

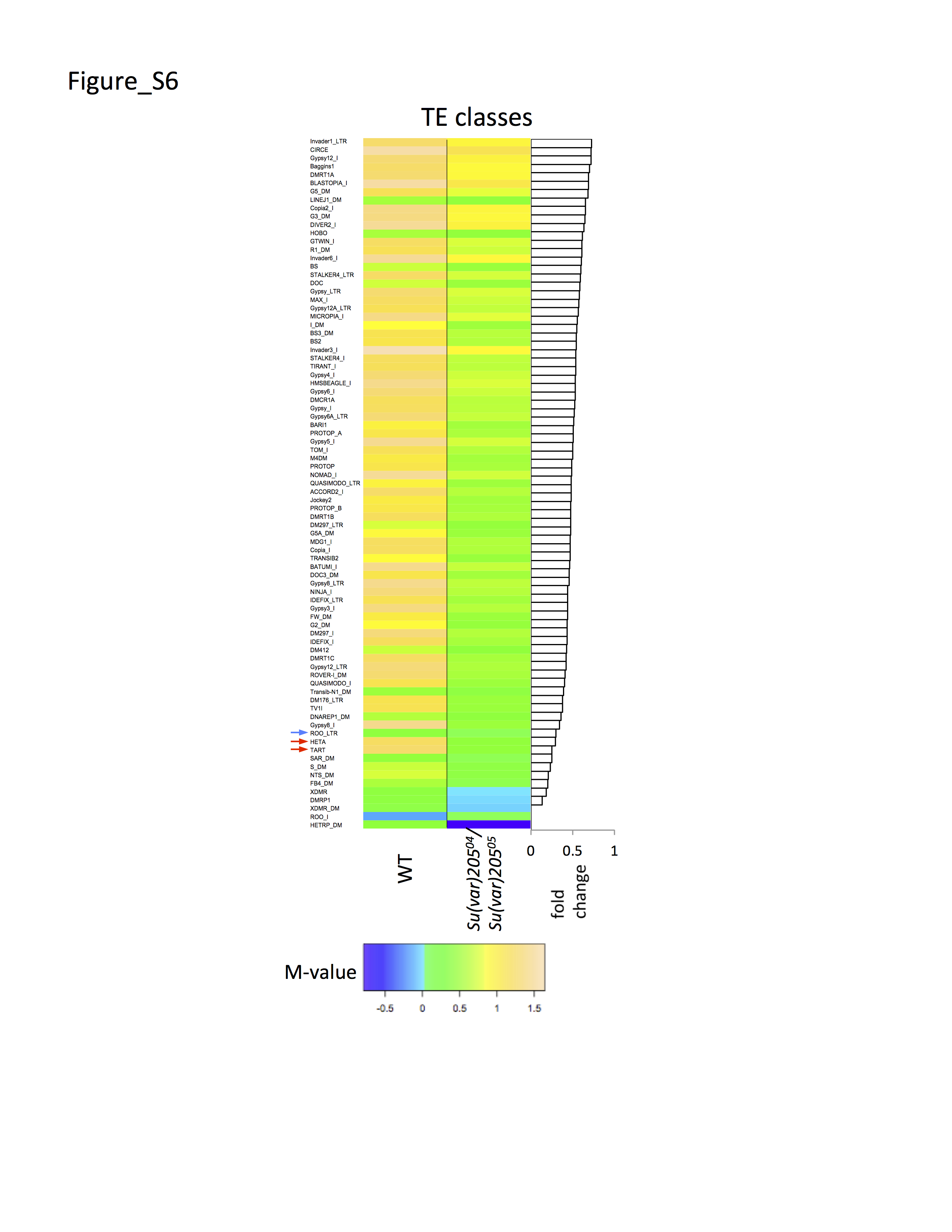

Supplement: Figure S6 — H3K9me2 enrichment profile in various TE classes in Su(var)20504/Su(var)20505 mutant larvae. While absolute values vary, loss of HP1a consistently leads to a loss of H3K9me2. WT = wild type; Su(var)20504/Su(var)20505 = HP1a depleted mutant larvae. (TIFF) [file pgen.1003780.s006.tiff]

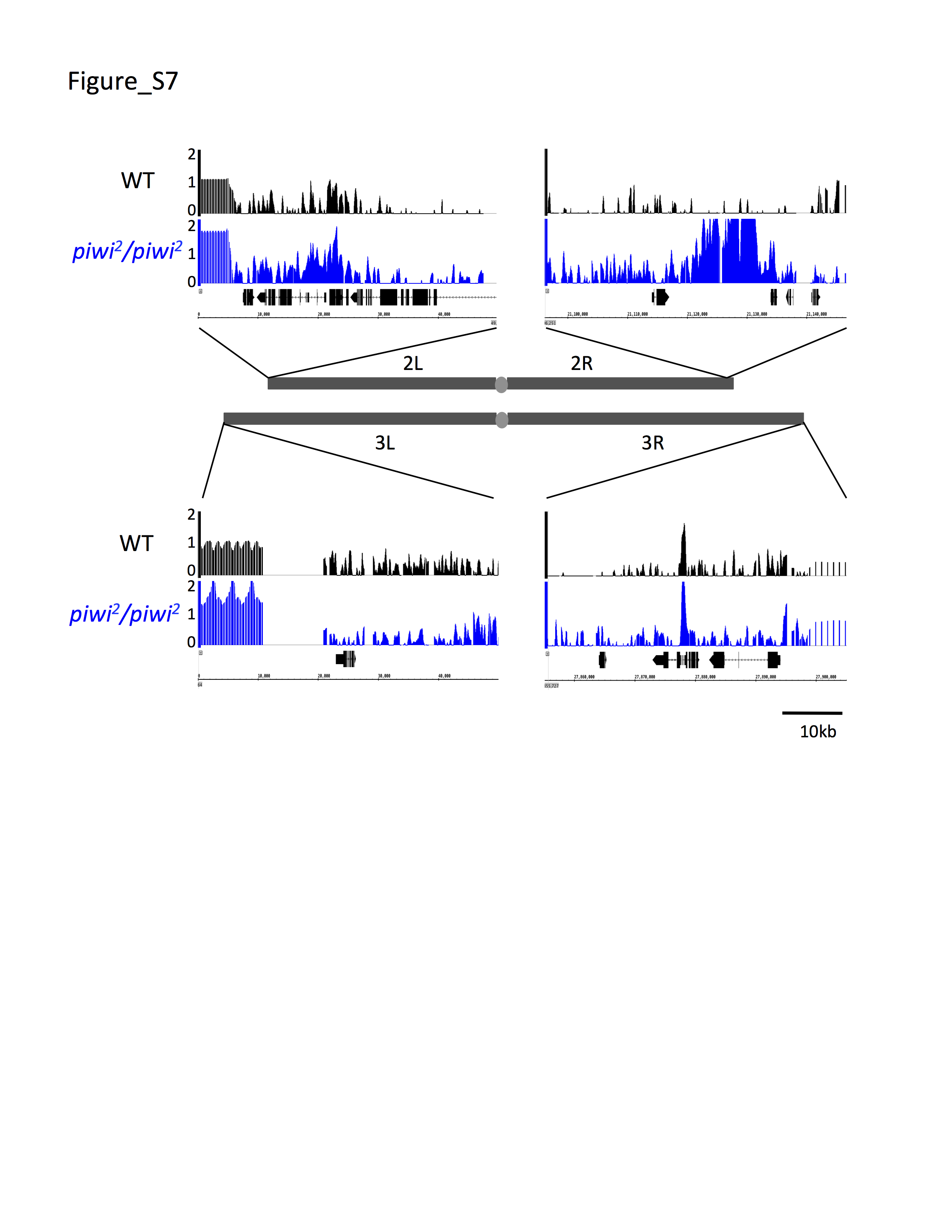

Supplement: Figure S7 — HP1a enrichment (M-value) in regions adjacent to the tips of the second and third chromosome arms in wild type and piwi2/piwi2 mutant larvae. For each chromosome arm, the 50-kb sequence adjacent to the end of the mapped assembly is shown. (TIFF) [file pgen.1003780.s007.tiff]

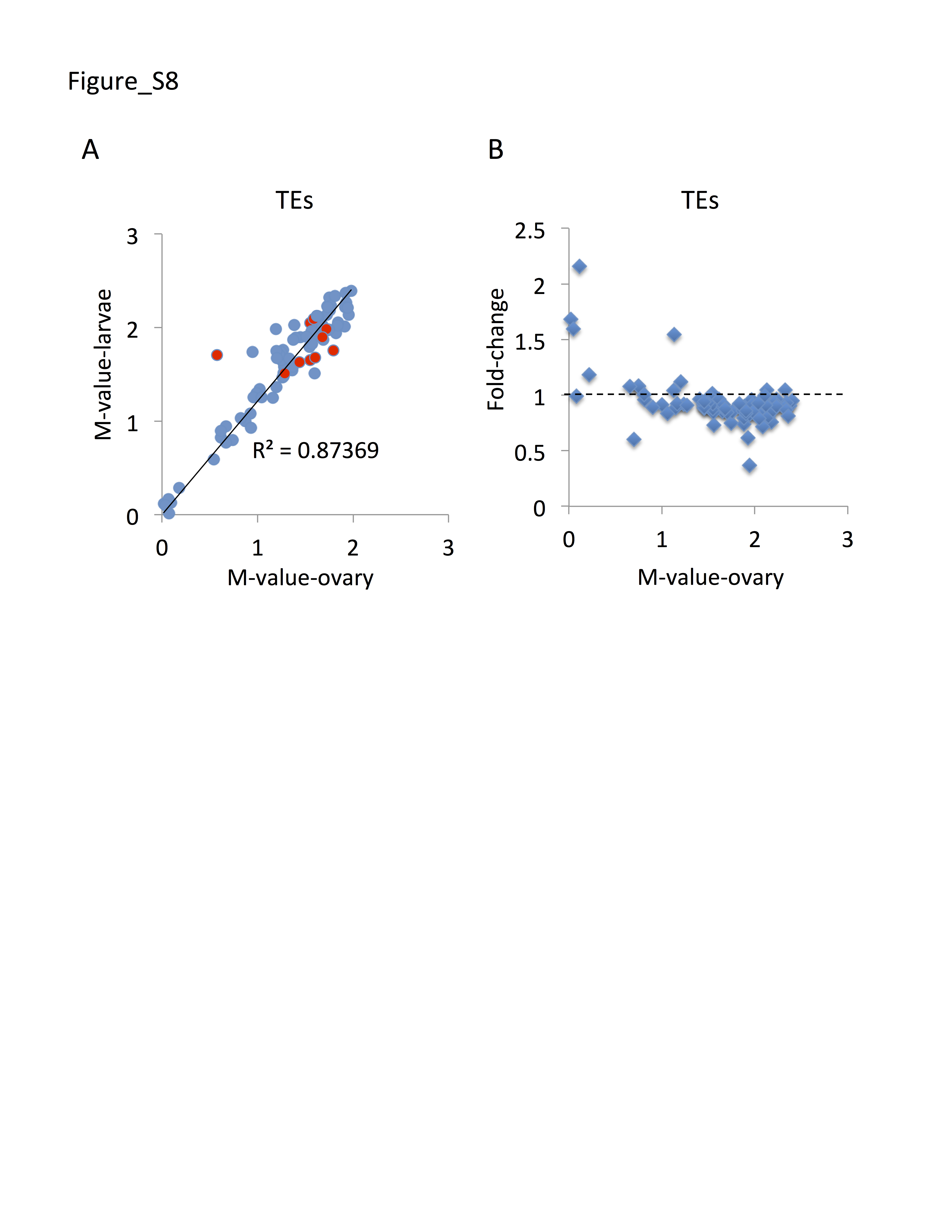

Supplement: Figure S8 — HP1a enrichment over TEs in female adult ovary. (A) The HP1a levels over different TEs are plotted, comparing ovary and 3rd instar larvae. Those TE classes showing the most HP1a reduction in piwi2/piwi2 mutant larvae are plotted as red circles. In general, the HP1a enrichment seen in ovary correlates with that seen in larvae. (B) HP1a reduction over TEs in piwi/piwi null larvae is not correlated with the HP1a enrichment in the ovary. The HP1a fold change in piwi/piwi null larvae (Y axis) is compared to enrichment levels in the ovary of wild-type adult females; no correlation is observed. (TIFF) [file pgen.1003780.s008.tiff]
