# Supplementary material for: Maternal Depletion of Piwi, a Component of the RNAi System, Impacts Heterochromatin Formation in Drosophila
Source: PLoS Genet. 2013 Sep 19;9(9):e1003780. doi: 10.1371/journal.pgen.1003780 (PMC3777992; doi:10.1371/journal.pgen.1003780)
Supplement: Table S1 — Fly lines used in this study. (DOCX) [file pgen.1003780.s009.docx]

Table S1. Fly lines used in this study.

| Stock number | Genotype | Source |
| --- | --- | --- |
| BL32564 | y^1^ w^*^; P{GAL4-nos.NGT}40; P{GAL4-nos.NGT}A | BDSC |
| BL5137 | [y^1^](http://flybase.org/reports/FBal0018607.html) w^*^; [P{UAS-mCD8::GFP.L}LL5](http://flybase.org/reports/FBti0012685.html) | BDSC |
| NA | *w^m4^*; *ey*GAL4 | Ahmid lab |
| BL33724 | y^1^ sc^*^ v^1^; P{TRiP.HMS00606.shRNA.PIWI}attP2 | TRiP |
| BL33400 | y^1^ sc^*^ v^1^; P{TRiP.HMS00278.shRNA.HP1a}attP2 | TRiP |
| BL34803 | y^1^ sc^*^ v^1^; P{TRiP.HMS00112.shRNA.EGG}attP2 | TRiP |
| BL34817 | y^1^ sc^*^ v^1^; P{TRiP.HMS00127.shRNA.G9a}attP2 | TRiP |
| BL34799 | y^1^ sc^*^ v^1^; P{TRiP.HMS00108.shRNA.AGO2}attP2 | TRiP |
| BL25710 | y^1^ sc^*^ v^1^ P{nos-phiC31\int.NLS}X; P{CaryP}attP2 | TRiP |
| NA | yw^67c23^; Su(var)205^02^ / CyO | Lab stock |
| NA | yw^67c23^; piwi^1^/ CyO | Lin lab |
| NA | yw^67c23^; piwi^2^/ CyO | Lin lab |
| NA | In(3L)BL1 | Eissenberg lab |
| NA | Tp(3;Y)BL2 | Eissenberg lab |
| ­NA­ | yw^67c23^; 118E10 | Lab stock |
| NA | Oregon R | Lab stock |
| NA | *yw^m4^*; | Lab stock |
| NA | yw^67c23^ | Lab stock |

BDSC: Bloomington Drosophila Stock Center; TRiP: The Transgenic RNAi Resource Project. All fly lines from TRiP are made in yw^67c23^ background before doing the pigment assays.
