# Supplementary material for: Maternal Depletion of Piwi, a Component of the RNAi System, Impacts Heterochromatin Formation in Drosophila
Source: PLoS Genet. 2013 Sep 19;9(9):e1003780. doi: 10.1371/journal.pgen.1003780 (PMC3777992; doi:10.1371/journal.pgen.1003780)
Supplement: Table S2 — Primers used for quantitative PCR. (DOCX) [file pgen.1003780.s010.docx]

Table S2. Primers used for quantitative PCR.

| Primer name | Sequence |
| --- | --- |
| Piwi_RT_forward | ACTTCCCGAGGTAGTGGTGA |
| Piwi_RT_reverse | CGGTTCCCTTCTTGGATACC |
| EGG_RT_forward | GTCTTCCAAGCAGCAGTTCC |
| EGG_RT_reverse | TCTGGCTGCTTCTCCTCTTC |
| G9a_RT_forward | TAGCGTTAAACTCGCTGCTG |
| G9a_RT_reverse | CTGAAGTGGGCGTGTCTATG |
| AGO2_RT_forward | CTTGTTGCAGCAGTTGACCTA |
| AGO2_RT_reverse | TCGGGGACAATCGTTCGCTTT |
| RPL32_forward | CGATCTCGCAGTAAAC |
| RPL32_reverse | CTTCATCCGCCACCAGTCG |
| lacZ_forward | ACTCACACACAATGCCTGCTAT |
| lacZ_reverse | GCA ACT GTT GGG AAG GGC GAT |
| hsp70w_forward | CAAGCGCAGCTGAACAAGCTAAAC |
| hsp70w_reverse | ATTGATGGCGTAACCGCTTGGAG |
| α-actinin _forward | cagcaagcacctctgctcta |
| α-actinin _reverse | tgcaagcgtatgtgagatcc |
| 18S_rDNA_forward | TTCATGCTTGGGATTGTG |
| 18S_rDNA_reverse | GTACAAAGGGCAGGGACGTA |
| HetA_P_forward | ACCACGCCCAACCCCCAA |
| HetA_P_reverse | GCTGGTGGAGGTACGGAGACAG |
| Blood_P_forward | TGCCACAGTACCTGATTTCG |
| Blood_P_reverse | GATTCGCCTTTTACGTTTGC |
| Baril_P_forward | CATGGGTCACAAACAGTTGC |
| Baril_P_reverse | TTCATTTGCCTCTTCCTTGC |
| light_forward | ACATTCAGGAGGCGTTGGTC |
| light_reverse | AGTTATCACCCACCTATTTTGC |
| rolled_forward | ACCACGACCTTATTTTACTTCC |
| rolled_reverse | ATGAAATGTGAGCGTGACGGTT |
| ci_forward | cagctcatcaacggcaaaca |
| ci_reverse | ccgcattggatttcattatcat |
| bt_forward | gcaaggacacaggcaaatacaa |
| bt_reverse | TCACAACCACCGTCATCATCT |
